# Stakeholder's experiences, expectations and decision making on reproductive care: An ethnographic study of three districts in northern Ghana

**DOI:** 10.1371/journal.pone.0186908

**Published:** 2017-11-01

**Authors:** Martin Amogre Ayanore, Milena Pavlova, Regien Biesma, Wim Groot

**Affiliations:** 1 Department of Family and Community Health, School of Public Health, University of Health and Allied Sciences, Hohoe, Ghana; 2 Department of Health Services Research, CAPHRI, Maastricht University Medical Centre, Faculty of Health, Medicine and Life Sciences, Maastricht University, Maastricht, Netherlands; 3 Centre for Health Policy Advocacy, Innovation & Research in Africa (CHPAIR-Africa), Accra, Ghana; 4 Department of Epidemiology and Public Health Medicine, Division of Population Health Sciences, Royal College of Surgeons in Ireland, Ireland; 5 Top Institute Evidence-Based Education Research (TIER); Maastricht University; Maastricht, Netherlands; National Academy of Medical Sciences, NEPAL

## Abstract

**Background:**

In Ghana, priority-setting for reproductive health service interventions is known to be rudimentary with little wider stakeholder involvement. In recognizing the need for broad stakeholder engagement to advance reproductive care provision and utilization, it is necessary to jointly study the varied stakeholder views on reproductive care services.

**Methods:**

We applied an ethnographic study approach where field data was collected between March-May 2015 in three rural districts of northern Ghana. Data was collected among women with recent births experiences (n = 90), health care providers (n = 16) and policy actors (n = 6). In-depth interviews and focus group discussions was applied to collect all data. Each stakeholder participant’s audio file was transcribed, and repeatedly read through to identify similar and divergent views in data. A coding scheme guided coding processes. All transcripts were then imported into QSR NVivo 11 for further analysis.

**Results:**

Four themes emerged. Women participants accentuated that sex and sexuality values of men have changed over time, and drives gender roles, parity levels and decision making on reproductive care needs at community levels. Sexual stigma on reproductive care reduces the willingness of women to voice poor experiences related to their previous reproductive experiences. All stakeholders’ highlighted clinical treatments for post-abortion care are minimally covered under the fee exemption policy for antenatal and postnatal care. Policy processes on service delivery protocols still is top-down in Ghana.

**Conclusions:**

Health teams working to improve sexual and reproductive health care must find suitable context strategies that effectively work to improve women reproductive care needs at their operational levels. Private sector participation and informal community support clutches are encouraged to advance the delivery of reproductive care services.

## Background

In sub-Saharan Africa (SSA), sexual reproductive health behavior of women is influenced by multiple individual and societal needs [1]. The double burden of early sexual debut and marriage within the SSA region exacerbates the need for timely sexual and reproductive health care [2, 3]. This is further enhanced by the rising unmet reproductive health care needs among women in the region [4]. Aside the health consequences of unmet reproductive health care needs, the economic burden of reproductive health is huge in most developing countries [5]. This economic burden coupled with adverse effects of reproductive health near-miss complications such as acute renal failure, hypertensive disorders in pregnancy among others aggravates long term reproductive care needs of women in later life. Global estimates show that $68 million to $76 million annually is needed to manage near-misses of unsafe abortion at facility level following unattained reproductive services needs in developing countries [5]. A recent review of unmet sexual and reproductive health needs in West African show that unmet reproductive needs are still high in countries such as Ghana, Mali, Senegal, and Benin [4].

In Ghana, priority-setting for reproductive health service interventions is known to be rudimentary with little wider stakeholder involvement [6, 7]. Recent evidence in Ghana shows progress on family planning access, knowledge and awareness for reproductive health care [8]. The recent Demographic Health Survey finds that sexual freedom of women aged 25–49 has increased [8]. Nonetheless, sexually active adolescents in Ghana are known to use contraceptives inconsistently, with lower reported levels of contraceptive use, 25% and 40% among girls and boys aged 15–19 respectively [9, 10]. Reported unmet need for family planning is high (29.9%). A recent study found that women have a high preference for covert contraceptive use. This is the case even among non-married women [11]. Preferences and intentions on the timing of child bearing and the desired number of children appear to drive reproductive decisions [12]. In northern Ghana, community level evidence on reproductive service uptake, such as family planning using the “Zurugelu Approach”, shows that introducing and sustaining reproductive care changes requires attention to the needs and concerns of multiple stakeholders. In particular, male spouse and hospital staff involvement effectively promotes family planning and counseling services. In particular, the involvement of the male spouse and the hospital staff effectively promotes family planning and counseling services [13]. But how exactly is the Ghanaian health system responding to the changing reproductive needs of women through information and counseling services? Recent studies in Ghana advocate improving women autonomy to increase reproductive health service utilization [11, 13–15]. These studies provide evidence of the social conditions, women preferences, and health system challenges in delivering reproductive health services. They are however limited in relation to stakeholders experiences and expectations, and socio-gendered role effects on reproductive decision making in rural Ghana. Knowledge on how health care provider’s experiences shape and influence women preferences and autonomy on reproductive care services use is also limited despite the growing consensus for broad stakeholder involvements in the provision and use of reproductive care services.

In recognizing the need for broad stakeholder engagements to advance reproductive care provision and utilization, it is necessary to jointly study the varied stakeholder views on reproductive care services. Reproductive care provision and use in this study includes user’s right to information, access to safe effective, affordable and effective strategies of birth control. Sexual reproductive health education, advocacy and reproductive health strategies for fertility control are included in this broad concept as well. Specifically, there has been growing need to understand the shifting expectations of user preferences on reproductive care services [16]. Priorities in policies on reproductive health are described in one study as ad hoc and limited [6]. This situation exists despite the increasing calls for public-private partnerships in the delivery of services. In addition, the question can be raised whether policy processes address user preferences for comprehensive, reliable and user-friendly services at the facility level as outlined in Ghana’s reproductive health policy [17]. In what directions do women perceive gender roles to influence their reproductive decision making and choices for care?

To identify these gaps, views of users of reproductive health services (women), health staff and policymakers were analyzed concurrently in three rural districts of northern Ghana. At the health policy level, this study provides stakeholder opinions of how women reproductive care shape reproductive behavior, choices, decision making, and priority-setting for policy decision making in rural Ghana. Our results add to the current knowledge on how various stakeholders’ roles, decision making and priorities impact reproductive health services provision and use in Ghana.

## Methods

### Study design

This study employed an ethnographic design. In ethnographic study designs, the voice of participants is an important source of data gathered throughout the investigation process [18, 19]. We choose to apply ethnography because we were interested in explaining the “culture” of the phenomenon using participant voice, behavior and observations from the field [20]. Ethnography allows for understanding the phenomenon from both the insider and outsider perspective [21, 22]. The design ensured that all stakeholder concerns surrounding the study aim were given a “voice”[23]. It provided the opportunity to examine the complexity of reproductive care needs and how the interactions of women, health staff and policymakers influence the provision and utilization of reproductive care services. Data were collected between March and May 2015 alongside another study that investigated stakeholder’s perspectives on shortcomings for maternity care in rural Ghana.

### Study setting

Three districts in addition to the capital of the Upper East region of Ghana form the study setting. The Upper East region is one of the 10 administrative regions in Ghana. The Upper East region currently has 13 administrative divisions under Ghana’s decentralization structure. Major ethnic groups in the region include Bimoba, Bissa, Buli, Frafra, Kantosi, Kasem, and Kusasi. The health system in the Upper East region is organized into a 4-tier system typical of most regions in Ghana; regional, district, sub-district and community levels often called CHPS centres. Three districts; Bongo, Talensi, and Nabdam were purposively selected for this study. The 3 districts were selected due to poor indices of maternal and reproductive health outcomes among the 13 administrative divisions in the region. Proximity and resource availability for data collection also influenced choice of districts. In each of these districts, three remote facilities providing reproductive health services in each district were selected (9 facilities in total). We also included Bolgatanga, the capital of the Upper East region as one of the settings because some policy interviewees resided in this area.

### Data collection and sampling procedures

Data were collected among three groups of stakeholders: women with records of recent births (2 years prior to study), health staff, and policymakers. Focus group discussions (FGDs) were conducted with women and health staff while in-depth interviews (IDIs) were held with policymakers (public and private). To select women participants, midwives in the included facilities provided information on 15 women per facility with recent birth experiences using data from antenatal and postnatal facility registers. In total 135 women were selected as potential participants in all 3 districts. At each district level community, meetings were held across all 9 facility settings to select final women participants for the study. A final inclusion checklist with the following criteria was used to rank final participants at each facility: woman should be physically present at meetings, consent to participate, interested in the study and able to share prior knowledge related to the study aim. At each facility meeting, 10 participants with high rating towards study were selected. A convenience sample of 30 eligible women per district took part in the FGDs (3 FGDs per district with 10 women in each FGD). Thus, in total 90 women took part in the study. Each woman participant was provided a participant consent form and was asked to complete this form through signing or thumbprint before all interviews started. Across the three facilities per district, health staff in one of the facilities was selected. In these district facilities, one midwife, a senior staff in-charge of facility, and facility nurses (on average 3–7 persons per district) responsible for providing reproductive health services at these facility centers were recruited as district health staff participants. Policymakers were later recruited and included 2 private health policy program managers and 4 public sector policymakers. The two private health policy managers worked in health facilities under the umbrella Christian Health Association of Ghana (CHAG) across our study settings. All four public sector policymakers worked in public health facilities across study districts. All four policy actors read and signed a consent information sheet before the start of each interview. FGDs with women were conducted in one main district local language; Talensi (Tali), Nabdam (Nabit) and Bongo (Grune). All IDIs were conducted in English. FGDs and IDIs were conducted using sample structured guides (see Table 1).

**Table 1:** Sample structure of questions used to assess all stakeholder groups.

	*Reproductive health care experiences*, *expectations and policy process on reproductive health care needs*
a.	What experiences, expectations do you have with regards to using/providing reproductive health care? Probe further on social, gender and environmental drivers for women independent life choices in reproductive health care.
b.	What experiences underpin reproductive preferences/needs among users and providers in the facility/districts level? Probe further on challenges, barriers that affect your ability to deliver basic and comprehensive reproductive care services at the facility/district level?
c.	What expectations define the reproductive preferences in provision (health staff, policymakers) and its use (women)? Probe on how each stakeholder meets and unmet expectations.
d.	What priority setting informs reproductive health choices and preferences in facility/district? Probe on concerns policy decision making process have for users in rural settings. Probe further on policy standards in use and what they seek to attain in reproductive rights issues.
e.	How does policy processes address user preferences for reliable and user-friendly services for women, health staff and policy makers. Probe further on services delivery mode, previous experiences impact on current reproductive needs? Follow up with probes on reproductive policy implementation processes and inclusiveness and how this relates to meeting user reproductive care preferences.

In conducting the interviews, the ethnographic approach allowed for continuous inductions and verification of all stakeholder views. FGD and IDI audio files were transcribed verbatim into English by two research team members (the principle researcher and an assistant). We used two research team members to minimize single level biases in our analysis. Transcripts from all stakeholder groups were repeatedly read by the principle researcher and the assistant to identify similar and divergent views in data. All individual stakeholder transcripts were finally checked for accuracy and consistency with the original audio files. The research team then developed a coding scheme to guide the coding for each stakeholder group response based on the aim of the study.

### Data analysis

To ensure consistency among the two coders, we developed coding rules to facilitate the process. An initial coding for transcripts was undertaken by the principle researcher and the assistant using the principles for open coding [24]. All transcripts were then imported into QSR NVivo 11. In NVivo, one research team member undertook a further induction (open coding) for stakeholder concepts while another team member audited the first coder work to ensure consistency and reliability of coding in NVivo. Each imported transcript was analyzed in NVivo separately. Constant comparison enabled a further reduction of concepts, codes and nodes into themes [25]. To show how individual stakeholder views relate to each other and our study aim, we used the model explorer tool to map out how each stakeholder themes relate to each other [26]. All codes were further refined until we reached saturation. To guarantee the reliability for our results, we estimated interrater coding reliability for all coded data. Women, health staff and policymakers recorded r = 0.98, r = 0.96 and r = 0.94 respectively. Mapped out results for all stakeholder themes are tabulated and further illustrated in the results section.

### Ethical consideration

All participants were provided with detailed information about the study and how data are collected, used and stored in the study. During the data collection, we ensured that accurate and adequate data on the study aim was captured. We also ensured strict compliance by all research team members to prevent unauthorized access to participant’s data, loss, destruction or damage to any data collected. Study approval was granted by the Institutional Review Board of the Navrongo Health research Centre, Ghana with ID number: NHRCIRB202.

## Results

### Characteristics of participants

Parity levels of the 90 women in the FGD ranged between 1 and 7 births. Only 13 women ever had a home birth. Four women reported pregnancy miscarriages. Six women reported they ever sought post abortion care. Two women indicated they had to pay for post-abortion care services. Ever use and discontinuation of contraceptive use was reported by 18 women. Few women however knew where to obtain related services such as sexual transmitted infection (STIs) information and treatment. No woman reported to have been examined for any related reproductive cancers. The health staff was represented in the study by 3 midwives, 3 senior health nurses and 10 junior community/enrolled nurses with varied levels of working experience within the Ghana Health Service. Policymaker’s participants were responsible for policy dissemination at facility, community, district and regional level.

### Findings

Four main themes emerged from the analysis in NVivo: 1) gender roles on reproductive care needs, 2) the experiences on meeting reproductive care needs, 3) the expectations about reproductive care needs, and 4) the policy setting and decision making processes on reproductive care needs. Relevant abstracted findings based on the 4 themes are presented below. The results highlight comparative synthesis of participants’ views showing consensus and aberrations across each stakeholder group.

### Gender roles and reproductive care needs

This theme emerged only among women participants. Table 2 presents women’s opinions of how gender roles drive their reproductive needs from early adolescence to later life. Gender role findings show women belief that external friendship ties among others could assist them to meet their reproductive choice need even if they suffer opposition from the family support. This is particularly true for women who use contraceptives covertly. At the community level, the majority of women accentuated that sex and sexuality values of men have changed over time partly due to economic roles played by women at different levels within the family. Women accentuated that male perception of their domineering power over women drive parity level decisions, gender roles and fertility decision making on reproductive care needs. Another highlight of women expressions is that social perception and attitudes on decision making favors male spouses more than women. Some women opined their resolve to covertly use certain reproductive health services at the facility level if the spouse does not recognize their reproductive needs (Table 2 w^5^). However, some women’s views show that their ability to economically support male spouses to meet family care needs, gives them leverage to covertly meet certain reproductive care needs (Table 2 w^3^).

**Table 2:** Women views on gender role impacts on attaining their reproductive care needs.

Theme 1	Women accounts
Gendered role impacts on reproductive care	“Of choices, we often have them before we arrive at the facility. But sometimes we make these decisions with other women and peers since we cannot discuss these issues with our husbands”-w^1^.
	“we value our social norms on fertility too, so we often struggle to balance this with reproductive services that demand we stop childbearing”-w^2^
	“Our society requires male spouses to dictate how and when we should have sex and pleasure, but women ability to support as breadwinner makes other household decisions helps us sometimes have autonomy for our childbearing needs.”-w^3^
	“some women covertly use contraceptives to avoid conflict at home, and keep men responsive to their needs”-w^4^
	“Our male spouses have power to demand sex we cannot deny, but we too have power to deny the effects when we use family planning services provided us at facility.”-w^5^
	“Although people advocate for male spouse to limit or space births, we will be happy if the conversation should be asking males spouses to respect our individual psychological needs because childbearing puts so much stress on us and our children…”-w^6^
	“advocating for us to make our birthing and postpartum services inclusive with our male spouses will be problematic because we need to take good decisions concerning our well-being”-w^7^
	“Health staff just listen and ignore our problems on clinic opening times, sometimes when you live far and would wish to hold you in a holding bay until your labor starts….”-w^8^
	“They shout when we are going through pain at childbirth, simply because your life is dependent upon them ….such experiences will inform us change different facility or consider friendlier home care using a traditional birth attendant…”w^9^
	“I just obey what the nurse say I should do without questioning at antenatal, otherwise, you are skipped since so many women are waiting for the same service”-w^10^
	“Male volunteers help us a lot, always willing to listen and refer us to nurses when we have concerns on reproductive services”-w^11^

W^n^ -W represents women views and superscript indicates number of record presented in table

Gender roles in this study also showed women expression of health staff cluttered controls over women at health facility centres. Some women indicated that they had been skipped or ignored by the staff, or received insufficient attention by nurses during post-partum family planning care. This often happened if health staff perceived that women challenged the status-quo when receiving reproductive services as asserted by women. Some women also expressed that were skipped during antenatal visits by female nurses as expressed in Table 2, w^10^. When male health staff power relations were quizzed, women indicated that the impact of male nurse staff on their reproductive behavior was limited. The majority of women however indicated that male community volunteers assisted and provided them feedback on addressing reproductive needs (Table 2, w^11^).

Furthermore, women views on care and support beyond reproductive services provision (family planning injectables and other antenatal services) at the community level were varied with that of health staff. Women were of the opinion that services delivery on reproductive health did little to address psychological aspects of care. Most women accentuated that services, which address their emotional and physical self-worth concerns, were absent in most facilities they often seek care. In the absence of psychological support, strong family opposition to women making independent reproductive choices has an impact on women’s preferences. Women indicated that negotiating in case of familial opposition is often difficult (see Table 2, w^6^).

### Experiences relating to reproductive care needs

The experiences-related theme was observed among all three stakeholder groups (see Table 3). User experiences among women enumerated here relates to the lack of reproductive services non-differentiation for users, the cost implications for post-abortion care, and the social stigma associated with demanding abortion care services. Health staff stated that their inability to deliver multiple reproductive services is because most clinical reproductive services are not costed and financed thoroughly. This was seen by women as a deterrent for visits to community health facilities for reproductive services (Table 3, hs^1, 4^).

**Table 3:** Stakeholder experience with service provision to meet user reproductive preferences.

Theme 2	Women	Health Staff	Policymakers
Experiences relating to meeting user reproductive needs	“health staff provide services in a general form, without differentiating our individual sexuality states and preferences”-w^1^	“Many reproductive health services outlined in Cairo are not delivered; we cannot diagnosis conditions such as reproductive cancers and offer post abortion services in this facility or the district. A woman has to go to the regional level”-hs^1^	“Some health facilities started in already existing structures originally designed for other purposes. Some users assertions of poor clinic facilities arrangements and privacy issues are been considered in building new facility infrastructure”-p^1^
	“My last experience with induced abortion care was really worrisome…I lost my baby and was asked to pay (USD 25.00) in order to clean my womb. Whiles we are told childbirth care is free under free fee user policy”-w^2^	“In instances where we cannot address women reproductive need, we just refer them to the district”-hs^2^	“At the community facility or district facilities, post abortion services are not provided…supplies to undertake post abortion caring services are not covered under the fee exemption ANC/ Postnatal policy under the health insurance, these charges will need to be accounted for if rising cost at the higher policy level”-p^2^
	“Nurses contribute to some women unwillingness for contraceptive use because of poor communication…”-w^3^	“We have yearly plans we submit to our facility in-charge and to the district level on reproductive services, the money is just not available to do a or be that you have outlined to do”-hs^3^	“We acknowledge most of our services are stigmatized. Users seeking abortion care, HIV/AIDS, STIs and even fertility treatments are perceived as ill intended by general populace, private providers providing these services are even labeled in the public eye”-p^3^
	“I stopped going to my community clinic, over three years, they have always have one type of family planning products”-w^4^	“Our targets on post-partum family planning is usually not continuous, hence many barriers for women use after birthing practices”-hs^4^	“Most stigmatization issues may results in underestimating women experiences, something the formal health system must design structures to address”–p^4^
	“When my daughter got pregnant whiles in school, we were traumatized and stigmatized because we are also insulted and mocked when I come with her to the clinic for antenatal care.”-w^5^	“We have facility procedures to engage users and local actors on better addressing their needs.”-hs^5^	
	“Services are delivered in one facility room, young women, adolescents. This does not make them friendlier since most young people do not come whiles teenage pregnancy is rising”-w^6^	“I think it’s still problematic in delivering integrated services because many service needs in reproductive health are not supported in facility level expenditures”-hs^6^	
	“Societal stigma and name calling for our girls who become pregnant in the course of their studies is a challenge, and we will prefer they are provided with family planning services too because they will all grow soon as mothers”-w^7^	“we never receive women complaints on unmet preference needs, we rather hear of these complaints outside the facility level”-hs^7^	
		“there are certain factors that goes beyond us, such as women demands for home visits when they choose to deliver at home”-hs^8^	

W^n^, hs^n^, p^n^ denotes women, health staff and policymaker’s expressions. Superscript ***n*** denotes the number of view counts expressed by participants.

Women asserted that the health staff did not differentiate between adolescents and women with higher parity desires (Table 3, w^1, 6^). Women reported a lack of facility space for services related to infertility counseling, and STI diagnosis for women. Views among health staff and policy makers largely show that most public health facilities are unable to diagnose conditions such as reproductive cancers and offer post abortion services in this facility or the district (Table 3, -hs^1^). Women (n = 10) who experienced pregnancy terminations (induced abortion or miscarriage) paid high fees to obtain post-abortion care needs, which was never a rewarding experience. A woman recounted paying USD 25.00 to obtain post-abortion care services (Table 3, w^2^).Policymakers in responding to post-abortion fee cost indicated that clinical treatments for post-abortion care were minimally covered under the fee exemption policy for antenatal and postnatal care (Table 3, p^2^). Policy makers and health staff support women suggestions that abortion and post-abortion care services should be a part of the care continuum for reproductive health services.

Additionally, poor women-health staff communication was observed to contribute to the unwillingness of women to use contraception as highlighted by women (Table 3, w^3^). Few women reported that the health staff’s unaccommodating attitudes towards young women and adolescents seeking abortion care, lowered the trust among new users.

Specifically, women mentioned they have been traumatized by bad experiences when they accompanied their young adolescent girls for antenatal care (Table 3, w^5^). Although health staff did not respond directly to women perceived views on women-health staff relations, they expressed their views that they undertook their duties in conformity with facility procedures for engaging clients who visit for services (Table 3,”-hs^5^). In instances where health staff cannot meet women expected reproductive service preferences, users and local actors are often unable to explain what services are better met at community facility centres (Table 3, hs^5^).

On how women-health staff relations are better managed to improve quality of reproductive services delivery at the policy level, policymakers and health staff both agreed that health facility level evaluations of staff performance are one way to monitor reproductive and other general services delivery. Women agreeing to this however indicated that these feedbacks of evaluations are sometimes not immediate, often exacerbating trust and relational issues between health staff and users at facility levels. Never the less, policymakers reported periodic community engagements help to understand user’s preparedness and demands for quality and satisfactory service needs.

Policymakers recognized that new health infrastructures prioritize user needs, such as adolescent reproductive friendly centres spaces to cater for individualized needs in new public facilities. The need for privacy to reduce the negative stigma among intended users, particularly counseling for HIV/AIDS services has improved (Table 3, p^3^). Social stigma on the use of some reproductive services often contributed to negative experiences with reproductive demands at facility and community levels as agreed by both women and health staff. Stigmatization may sometimes reduce the willingness of women to voice poor experiences related to their previous reproductive experiences.

### Expectations on reproductive care needs

The expectations-related theme was catalogued based on all three stakeholder groups. Women’s views here reflect assertions that the health system has overly emphasized and relied on products and services to meet their reproductive health needs. Examples of how facility level structures meet or fail to meet women’s needs are reported by health staff and policy makers. At the policy level, what needs to be done more to meet user needs was highlighted by women discussants. Women views expressed reflect their expectations for more information sharing by health staff in meeting health effect concerns, particularly among contraceptive service users. The majority of women indicated they are hardly provided with adequate and appropriate information on how to overcome negative side effects. Services were more focused on family planning with little attention paid to sexual disease prevention, opportunistic infections such as reproductive cancers and infertility treatments (Table 4, w^2 & 3^). Health staff indicated that comprehensive reproductive care can be met when several skilled para health staff is available to provide services (Table 4, hs^6^). Health staff attributed health system inability to meet this demand as partly accounting for these concerns raised by women. Policymaker’s views substantiated the health staff perspectives, emphasizing services such as shortcomings in abortion care, infertility treatment, and physical infrastructure deficits, which have to change dramatically to meet user preferences (Table 4, p^1 & 2^). Even when reproductive health services provided are desired by a user, the inability to ask questions and be provided feedback on appropriate method use limit meeting women desired contraceptive needs. A women recount a health staff rhetorically saying “*I couldn’t do anything”*. This was agreed by many women as a common response when they push further on meeting certain expected services at the facility level (Table 4, w^4^).

**Table 4:** Stakeholder expectations on provision and meeting women reproductive care preferences.

Theme 3	Women	Health Staff	Policymakers
Expectations on reproductive care needs	“At first we use to think more of family planning, now we will prefer health personnel talk more also on sexual disease prevention because of recent diseases we hear of these days”-w^1^	“some stuff do not know all the components of the Cairo targets on reproductive health…they are basically involved in family planning and few components of antenatal and postnatal care”-hs^1^	“Improving staff skills is something the ministry and at our level we continue to undertake. We acknowledge current inabilities to provide services such as abortion care, post-abortion, and fertility treatments mostly in the public sectors”-p^1^
	“We are always told of contraceptive side effects, but they don’t give us appropriate information on how to overcome or address these issues”w^2^	“even when we know what women need, can we meet that simply by knowing, more has to be done from the Ministry of health to support in early diagnosis and treatment such as opportunistic infections on STIs, reproductive cancers, and safe abortion care”-hs^2^	“What we need to do more in meeting user expectations is to invest more in infrastructure. At our policy level we acknowledge the difficulties although we cannot drive this big policy push”–p^2^
	“We always want opportunity to ask questions most times this is difficult because some nurse don’t understand our language when we explain”-w^3^	“I did not know we are expected to provide infertility services, I always thought it was the role of the private sector in such services”-hs^3^	“The health centres or district hospitals although some staff have received training cannot even provide safe abortion services, this sector has entirely been taken over by the private sector where limited people may access services because of cost and stigma”-p^3^
	“I did have concerns about the health effects of my family planning method choice, after complaining to the nurse, she just asked me to cope since she couldn’t do anything”-w^4^	“Sometimes we have difficulty dealing with women own difficulties when we don’t know the appropriate health counseling demands to meet their need”-hs^4^	“Helping women manage health consequences is a key component of our counseling services. One of the greatest challenge is addressing meeting individualized focused preference needs where health staff are limited”-p^4^
	“When they want to meet us to provide services, they just inform us through a volunteer, forgetting we have we need convenient times to better use services… we use to be fine but working demands making some women unable to attend these sessions from health staff”-w^5^	“Sometimes women encounter psychosocial problems; they come to us instead of a psychologist. We can do little in this regard to support them, we need health psychologist to help provide care for such women”-hs^5^	“Because of community engagements with staff, users and using community volunteers who communicate our messages to build trust among users and the health care system. These strategies are aimed at improving user complaints on unfriendly health staff and often unsatisfied conditions”-p^5^
	“I won’t go out to the child welfare center for reproductive services, the place is not only open but services are supported by non-experienced community volunteers”-w^6^	“comprehensive reproductive care can be met when several skilled staff provide services, women do not recognize that and assume facility level staff alone should be able to meet all their demands at all time”-hs^6^	“Contextualization of polices for health staff go beyond meeting user expectations to disseminating standards of care”-p^6^
	“An NGO working here few years ago made it possible to have community volunteers that visited us to at home after childbirth to give our child an injection…this has changed since we need to travel long hours in the sun with my children…”w^7^	“Most of the reproductive targets set up in the country reproductive policies have not been contextualized for us, and no adequate training for health staff. Contextualizing would enable professional psychological care for intended user’s”-hs^7^	“The difficulty has always to do with who disseminates these policies/protocols and how much we are committed to spend on improving continually these standards together with providers to meet user expectations”-p^7^
	“Our mother-to-mother support groups are supportive. We wish health nurses learn how to incorporate this as part of their outreach services”w^8^	“informal groups such as mother-to-mother support groups spoken by women is helping us address myths and misinformation on reproductive services offered at facility level, we support such efforts since it promotes our work as nurses”-hs^8^	“we are working to make informal structures and groups at community level more involving, accountable and supportive to address users expectation at our facilities”-p^8^

W^n^, hs^n^, p^n^ denotes women, health staff and policymaker’s expressions. Superscript ***n*** denotes the number of view counts expressed by participants.

Additionally, the demand for more organized integrated services rather than visiting the facility severally for different reproductive services is reported by women. Health staff responses on integrated services show the lack of knowledge about women’s basic infertility needs by health staff and the lack of facility level infrastructure add to the inability to meet integrated reproductive services for women. Policy makers cited individualized counseling as a great challenge to health staff, despite improvements in skills training on the job for health staff (Table 4, hs^4^). Health staff views showed that some women rely on the few available private medical centres to meet such basic service needs. Some health staff agreed partly to these women expressions of unmet reproductive expectations. This was driven by health staff assertion that they lacked appropriate counseling skills beyond family planning (Table 4, hs^1^).

Other expectations’ concerning the delivery of reproductive care services is related to emotional and psychosocial care for users and how the health system is supporting meet this need. Women recounted timely information sharing by health staff to influence their reproductive care choices should be a top priority among service providers. In addition, health staff and policy makers agreed to women views of the need to improve social support and care on reproductive services provided at facility levels. Women views indicate health staff’s inabilities to address their psychological needs create low self-esteem for intended users. This impacts women’s abilities to realize their desired fertility choices. Health staff avowed that unmet women reproductive psychological needs are not prioritized in meeting reproductive care needs at facility levels (Table 4, hs^5 & 6^). Both health staff and policy makers affirmed that the contextualization of most reproductive health targets would improve expectations of psychological support for reproductive care (Table 4, hs^7^ and p^6^). In addition, women again alluded to expectations of experiencing services delivered to them at closer proximities and at their home. Health staff expressed the view that local level arrangement at the facility level tailored towards increasing patient-health staff contact hours, so as to address more user complaints and unfriendly relations often reported by women is one of the ways to improve patient-centered reproductive care needs (Table 4, p^5^). A broad consensus on the existence of informal groups and their role in influencing the use of reproductive health care services at community level was asserted by each stakeholder participant. Policy makers recognized the growing concerns about informal social support at some community levels, and their active engagements with some health staff to provide them community based reproductive counseling services. More importantly, policy maker’s views indicate the need for health system to be more accountable and socially supportive to meet reproductive needs (Table 4, p^8^).Women views on informal group structures supported policy makers expression for the formal recognition and adoption of informal social support groups such as *mother-to-mother support groups* to provide them formal health advocacy information and psychological support. Health staff views supported women and policy maker assertions, indicating that informal group structures such as the mother-to-mother group’s help to address myths and misinformation on reproductive services offered at facility level (Table 4, hs^8^)

### Policy setting and decision making processes on reproductive care

This theme emerged from varied expression among the 3 stakeholder groups in the study. Policy maker’s consensus on policy setting and reproductive services provision supports that most policies are driven by donor funding. Hence most decision making priorities align to who provides funding and to which areas funders allocate their funds (Table 5, p^1^). Policy implementers and program managers only have the opportunity to prioritize a few activities from reimbursements received from the National Health Insurance Authority as reported by public policymakers (Table 5, p^4^). Most activities prioritized at this level are for reproductive advocacy (Table 5, p^5^). Health staff views on policy setting show that they sometimes do little to influence reproductive decision making at policy level. Health staff asserted that most policies just come from the top, sometimes neglecting women pressing reproductive needs at the facility or community level. Health staff recalled how refresher trainings are the only periods they are introduced to reproductive service or products (Table 4, hs^2^). The policy processes only involve health staff when they have to disseminate skills for service improvements as policy makers make an argument (Table 5, p^6^). Women on the other hand in two of our study districts acknowledged that they have been asked to participate in health program manager’s advocacy meetings in the past, but few policy inclusion changes have occurred. Women posited that although they have provided suggestions on how health staff and policy makers can better address their reproductive needs, they have become tired of these policy-provider engagements since their inputs are minimally incorporated into the services delivery (Table 5, w^1,2, 4^). Health staffs opinions blamed policy implementers at the district level for doing little to improve women reproductive care needs (Table 5, hs^5^).

**Table 5:** Policy setting and decision making expressions in meeting women reproductive preferences.

Theme 4	Women	Health Staff	Policymakers
Policy setting and decision making processes on reproductive care preferences	“people come to the community to meet us and ask us question on how satisfied we are with services, but we never see any differences in what they provide now from the old”-w^1^ “some of us do not really care of who takes part in the decision making to deliver us services, all we want to see is more friendlier support from health staff, and our ability to use our health insurance to benefit from available services provided”-w^2^ “We have health committees at each community facility, just few members are often informed of the clinic facilities concerns for ore services”-w^3^ “onetime health staff and people from the district came and held a meeting with men on allowing us use facilities for childbirth, we were never involved although the aim of the meeting was to benefit us”-w^4^ “Our chiefs and opinion leaders play active role in passing information from health center to all of us, but that is not enough since we expect some more engagements from frontline services providers like nurses who attend to us”-w^5^ “Decisions on services and our expected roles as women when receiving these should be well disseminated using our women leaders to help us”-w^6^ “We see and recognize health staff as immediate providers of care, what happens in terms of how satisfied we are, relationship building and mutual trust affects decision making on meeting our reproductive preferences”-w^7^ “Although we do not determine what services facilities should provide, our decisions and choices based on certain service demands should drive providers and policy managers to take steps to make these services friendly and available even to the few women who need them”-w^8^	“At the facility, we don’t procure or singly adopt an intervention without prior authorization from the district or regional level”-hs^1^ “We are often called to the regional level for refresher trainings on reproductive health, something I don’t think can be considered as inclusive on how these interventions are designed”-hs^2^ “Most policies just come from the Ministry level, what is women pressing needs in Bongo here does not matter”-hs^3^ “most services are rendered based on health staff training received whiles in school, this is difficult to capture and meet changing needs of reproductive users”-hs^4^ “When women complain of preferences not met, we just tell them we are working on it… however that decision does not depend on us when the policy actors determine what service should be provided based on its availability to us”-hs^5^ “we just provide the services made available to us, if its counseling services, we provide what information is on the protocol”-hs^6^ “Some staff incorporates context need by engaging facility committees, but the challenge is these are not always supported in budget lines from district or facility level”-hs^7^ “Standards of services to provide are sent from the district level without prior sensitization and health providers inputs, we are only called for trainings when we complain of understanding and applying these standards in relation to the settings we work”-hs^8^ “Women have visited for services not present. we advocated for our capacities to be improved to provide these such as infertility, post-abortion care and building awareness on aspects of female genital mutilation, but these are not viewed important at the top people”-hs^9^	“Our priorities sometimes are largely defined by what donor money is available and what needs can be met at any particular time”–p^1^ “One major concern is so many private providers are active players in the reproductive health industry, but there appear to be little synergy between the public and private sector. Most of them are not fully captured, and involved in providing strategic services”-p^2^ “we sometimes prioritize, other times, funds are already allocated based on prioritized areas for the region”-p^3^ “activities that we often have the ability to prioritize are very few, so we do not determine which major reproductive activities in the district”-p^4^ “most of what we prioritize at this level has to do with education and sensitization activities”-p^5^ “Policy processes include health staff when they have to disseminate/apply skills for service improvements”-p^6^ “Reproductive, maternal and child health unit at district hospital levels was created to meet reproductive priorities. The challenge has always been too much focus on few streams of reproductive care.”-p^7^ “We should start the debate on how to fund these services, donors’ funds is been withdrawn in some service need…”-p^8^ “The Sector Wide Approaches (SWAp) MDGs, and now SDGs goals drive most approaches we do since funding comes along these lines”-p^9^ “The economic value of most interventions are lacking, so what should guide future policy decision processes…”-p^10^ “As private providers, we think the reproductive policy must set clear guidelines on reproductive policy settings standards, something currently lacking”-p^11^ “We have a monitoring and evaluation team at the regional level that ensures targets budgeted activities are undertaken”-p^12^ “I admit sometimes our activities are too ad hoc and sometimes we taking decisions at the district level do not understand them before we start implementing”-p^14^

W^n^, hs^n^, p^n^ denotes women, health staff and policymaker’s expressions.Superscript ***n*** denotes the number of view counts expressed by participants.

Another key highlight of policy setting processes and how this impacts on reproductive care among policy makers and health staff was the role of political leadership. Policy makers expressed the views that commitments at the socio-political level have driven major reproductive care needs over the last decade (Table 5, p^6^). Some policy makers recalled how the Sector Wide Approaches (SWAp) and Millennium Development Goal (MDGs) targets have made contraceptive services available in some districts. SWAp in health were developed in the early 1990s with an aim to respond to widespread dissatisfaction with fragmented donor-sponsored projects in Africa. Ghana health sector SWAP formed the basis for investments and actions by the Ministry of Health and donor partners. Despite shortcomings with SWAps, the approach has been shown to have supported alignment, harmonization and improved accountability between donors and country governments who adopted the approach. The MDG period in Ghana also witnessed reproductive interventions in the MDG Accelerated Framework (MAF), reduction of maternal mortality, comprehensive antenatal clinic programs, safe motherhood, and prevention and management of safe abortion programs. A challenge admitted by policymakers is the poor application of economic evaluations to inform future prioritization of reproductive care services (Table 5, p^10^). This offers a challenge in estimating effectively on-going targets for reproductive health interventions at the public-private health sector levels. Private health policy makers view support the need for improved guidelines on reproductive policy setting standards for effective delivery of services by health staff (Table 4, p^11^).

Women expressions reflect little involvement of community level actors and leaders in policy setting and decision making (Table 5, w^5^). Women indicated their decisions and choices based on certain service demands should drive providers and policy managers to take steps to make these services friendly and available even to the few women who need them (Table 4, w^8^). Health staff Health staff stated that the decision making for protocol dissemination of reproductive services has little involvement with facility level staff. Women views also indicate they had no ideas on what standards and protocols dissemination plans are used in providing services for them (Table 4, hs^8^). Both women and health staff views reflect the need for better information flow and provider centered training needs to better address user concerns and needs. Overall, women recognized that their involvement in reproductive policy decision making was limited. Women views show their high positive ratings on friendly and prompt reproductive services compared with their direct involvement in policy setting for reproductive care at the facility level. Health staffs observed that their influence on women reproductive care choices is based on conditions that influence services use at the facility level. Most health staff admitted that some services are largely not provided because of logistical needs. All policymakers (public and private) strongly agreed that reproductive health targeting must be broad with clear guidelines to ensure reproductive care needs are properly contextualized.

## Discussions

This study has examined stakeholder’s views related to experiences, expectations, and policy decision making processes that influence reproductive care in three rural district settings in Ghana. In addition, women’s view of how gender roles influence reproductive care was unearthed. While women’s access to and use of reproductive care services may be influenced by broad social and economic determinants, the experiences, expectations, and policy processes enumerated by all stakeholders’ in our findings impact rural women reproductive care choices in rural Ghana. Overall, stakeholder consensus based on expressed experiences highlight the need for a more facility based resource allocation to meet facility based reproductive plans. Health staff views sharply differed from women assertions that services provided were non-differentiated. The reasons cited for this were that facility spaces and health staff were limited. Stakeholder’s opinions differed on what levels of counseling were adequate to meet user reproductive care expectations. Women viewed most counseling services as inadequate, and not tailored towards individual needs. Health staff and policymakers however were of the opinion that current counseling services were adequately designed, stressing that these counseling services were only limited due to low numbers of health staff to patient ratios at health facilities. To ensure current health sector policies on counseling meet user expectations, there is a need for consensus among key stakeholders on counseling components for reproductive care services. We discuss further under each results theme stakeholders views and how the policy processes meet or limit meeting user preferences for reproductive care.

### Gender role impact on reproductive care

Our findings on gender roles reflect women views on how gender influences reproductive health care in many societies in sub-Saharan Africa. Women’s views show that strong external friendship and ties outside the traditional family environment as a positive enhancer for their first acceptance and adoption of family planning services. Even in communities where health advocacy and education is provided by health staff, interfamilial associations influence women’s reproductive decision making and choice of care. Few narrations of households or communities where women lack economic power, fertility decisions are the preserve of the male spouse in consultations with other male external family heads. Even when it comes to birthing care, consultations in a traditional context require women acquiesce to final decisions prescribed by these traditional heads. Although many women admitted that male sexuality values have changed over time, this is limited to the family level where women’s contribution to economic livelihood is strong. Our findings corroborate several gender studies in developing countries that show that the women’s ability to exercise control at the household level improves their chances to use contraceptives and facility based birthing services [27–29]. Our findings show that male perceptions on sexual passivity of women drive male beliefs and desire to control women sexual behaviors. In situations where male spousal control neglects individual women needs, covert use of reproductive care services exist. This finding is consistent with other covert contraceptive studies [30–32]. Many women agreed that men’s role transects across abortion, pregnancy and childbirth, and infertility decision making. This corroborates anthropological studies that examined men’s multiple roles and influence on women’s reproductive health [33]. Women report that health staff often used cluttered control mechanism in the provision of services. This may indicate a structure of social networks existing between health care providers and users of health care services. Blanc (2001) distinguishes power in sexual reproductive relations where one form of power has negative and limiting authority over others ability to effectively utilize reproductive services [34]. In this study, health staff exercise of control and supremacy has the tendency to shy women away from meeting their reproductive needs. Systems and structures that create conditions of mutual respect among provider-user, and empower women in their healthcare seeking behaviors need to be encouraged.

### Experiences related to reproductive care needs

Our findings show that reproductive services non-differentiation at the point of service provision influences individual reproductive choices and use. There was consensus by all stakeholders on the need for service differentiation support the policy to provide reproductive services by taking into consideration the socio-demographic background of users and the group or social milieu of the intended user [17]. Services targeting general reproductive care in any population requires recognition and prioritization of different population group levels [35]. At the policy and provider levels, health staff and policy makers advanced views on the need to prioritize health infrastructure design and facility friendly centres as an entry point to facilitate the delivery of individualized reproductive needs. Overall, women reported fear of being stigmatized if services do not differentiate between elderly and younger women. In addition, stakeholder agreements from this study show social stigma could reduce user’s willingness to report poor reproductive experiences at the facility level. Women and health staff poor relational experiences regarding women use of contraceptives in the past may have been influenced by users perceived feelings they were socially insecure regarding contraceptive use. Most cluttered control perceptions held by women are associated with their non-involvement in reproductive service provided by health staff. Due to a high number of women who opt out for most reproductive and family planning services during specific clinic days, health staff rarely meet women’s individual needs during these contact periods. Health staff efforts to attend to women in such circumstances create provider-user relationships that are, often reduced to “calling and discharging” at the facility. Thus, women perceptions of cluttered control by health staff arises due to their non-involvement and health staff expectations that women do not to question any procedures or delays. These perceptions have a tendency to lower contraceptive use in an environment where the male spouse determines whether the couple uses contraception. This is also true in existing studies where social stigma is known to lower reproductive services use [36–38]. Existing studies support that adolescents girls have unique sexual care needs, are susceptibility to risky behaviors, and more likely to be exposed to inaccurate information on reproductive care services [39, 40].

Another important finding was stakeholders shared views on fee payments for post abortion care and management at the facility level by users. Women indicated that full fee payments were demanded before a patient is attended for pregnancy loss/miscarriage. Although some policymakers and health staff acknowledged this phenomenon, they sought to provide reasons why these demands exist. Policymakers and health staff views expressed here however did not indicate a justification of these experiences by women. Policy makers acceded to a need to fully include post-abortion care and management into the National Health Insurance policy benefit package.

Furthermore, health staff expressions on some facility level incapacity to provide a broad range of reproductive services is not helpful in addressing stigma and myths of family planning issues at the community levels. Health staff arguments points to the fact that in situations where reproductive services for STIs, abortion/post-abortion care, and infertility treatments are limited in scope. Myths on what define sexuality and womanhood in the society further foil social stigma on care seeking for such services as reiterated by health staff. Policymakers and health care staff attestation that some reproductive care services remain unmet and not currently provided remain more of human capital and finance need in Ghana. Health sector arrangements that diversify current and future reproductive human resource and financial needs will assist in many more services available for users.

### Expectations on reproductive care needs

One highlighted expectation by health staff and women was the need for health services to address reproductive opportunistic infection needs such as reproductive cancers and infertility treatment. Both stakeholder groups agreed that these services are mostly non-existent as part of service components offered. Private health providers play active roles in delivering a broad range of reproductive services absent at public sector level as agreed by policymakers and health care staff. However, since private facilities tend to be more urbanized, rural women seeking abortion and infertility treatments face distance barriers aside existing social stigma and norms for these services. Individualized reproductive care was also admitted by policymakers and health staff as difficult to attain partly to limited staff numbers and diagnostic facilities. This is documented as a major barrier in delivering maternity care services in Ghana [41–43].

Another repeated expectation among all stakeholder groups was the need to improve systems that are more integrative and supportive for reproductive service users. This study provides evidence that the non-availability of psychological support creates low self-esteem among intended users. While women expressed optimism that information and counseling enabled them to address clinical reproductive concerns, these counseling services were limited in supporting them to address individual psychological needs. The inability of the formal health system to address these specific psychological stress needs has a propensity to lower, or cause user withdrawal for several reproductive care services. Our finding here corroborates existing findings that a psychological burden is the most common reason for patients to withdraw from a facility treatment center for infertility [44]. In this study, health staff and policy makers accented that individualized reproductive care was difficult to attain, partly due to limited health staff expected to provide services. This reason could explain why most health staff have not been equipped and trained appropriately to provide other broad reproductive services such as fertility counseling and treatments and abortion care. Our finding that stakeholder’s are positive about delivering integrated reproductive services, and its associated barriers is consistent with other studies in Africa [45–47].

Women and health staff views were divided on how effective counseling services enabled users to attain their desired fertility goals. While women expressed that counseling services were limited in supporting them to address individual psychological needs, health staff were of the view that this challenge existed due to limited para health professionals to provide such services. The inability of the formal health system to address these specific psychological stress needs has a propensity to lower reproductive care services use. Our finding corroborates existing findings that a psychological burden is the most common reason for patients to withdraw from a facility treatment center for infertility [44].

Lastly, adopting participatory approaches for reproductive services delivery is acknowledged by all stakeholder groups as vital for improving reproductive services. The mother-to-mother support (MTMS) group evidence from our study draws more evidence of the important role played by informal community structures to promote health and well-being. Our study typifies studies across developing countries that support bottom-up approaches to delivery of community health services[48–50]. It also reflects evidence from other studies that specifically view informal groups as an integral component to providing satisfying health care needs in rural populations [51, 52].

### Policy setting and decision making processes

The policy processes for informed decision making on reproductive care services is argued largely by policy makers to be driven by previous and current donor funding needs. Health staff views align to top to bottom approaches on policy processes making it difficult for them to prioritize context reproductive care needs for intended users. Despite the health sector policies to ensure bottom-up planning processes, views from policy makers and health staff on reproductive care services agree to bottom-up planning gaps. Health staff at facility levels arguments was based on limited and delayed budgetary allocations for effective monitoring and reproductive health outreach activities in communities. Policy makers in agreeing to health staff indicates irregular insurance reimbursement further leaves most facilities ineffective in meeting basic to comprehensive reproductive service needs. An existing study on priority setting on sexual health in Ghana evidenced that reproductive policy setting is rudimentary, with little involvement from donors and advocates [6]. This supports top to down policy processes views expressed by all stakeholders in this study. While this study did not investigate donor funding sources and donor involvement in reproductive care, our results across all stakeholders indicate a weak involvement on reproductive planning process on products and services delivery among stakeholders.

Women indicated little involvement in policies on their expected needs, despite their involvement in health committee meetings with facility and district health providers. Agreeably, health staff also indicated most reproductive policy decisions come from the ministry, with little space for other stakeholder inputs. Health staffs influence on women reproductive care choices based on child birthing conditions at facility level, and not through involvement in policy processes on reproductive care. Health staff views impugned policy makers at district levels fail to recognize user concerns on policy processes on reproductive care services. Policy maker’s views differed with health staff assertions, indicating deficits in health sector funding and logistical needs account for perceived inefficiency at the district levels. Furthermore, policy makers agreed with health staff concerns that reproductive health targeting still remain limited in scope, since most reproductive care guidelines and protocols have not been contextualized and fully operational in all facilities.

Some major policy processes and decisions as argued by policy makers and supported by health staff and women have been driven by political commitments over the last decades. Specifically, many safe motherhood interventions implemented during the MGD period were influenced by political reform processes. One such policy is the free fee exemption policy for delivery care and the National Health Insurance Scheme in Ghana. These policies provided impetus for many health sector goals for improving sexual reproductive health needs in Ghana[53, 54]. Policy makers in our study noted poor application of economic evaluation to evaluate earlier SWAp processes concerning reproductive health interventions. This view corroborates exiting studies in Ghana that indicated misinformation and poor stakeholder understanding greeted some sector polices on sexual and reproductive health [6, 7]. Evidence from policy makers in our study also points to the poor application of economic evaluations to inform reproductive priority setting and decision making over the years. This suggests the need for reproductive systems to adopt approaches to measure track policy impacts over time to ensure policy process not only are inclusive, but contribute to improving reproductive health outcomes.

Overall, stakeholder views on the policy processes and its effects on user preferences for comprehensive and friendlier reproductive services show agreements centered on a top-down linear policy process and decision making. Largely, improvements in most maternal interventions have been made possible because of political commitment and other national and international agreements on improving sexual and reproductive health in Ghana. Despite this, gaps in meeting user individualized and psychological needs exist due to weak enforcement of the policy processes related to health staffing and health facility infrastructure needs. Reproductive health policy strategies that gives greater emphasizes on improving health staff capacity to contextualize reproductive working protocols at facility levels can help to meet user preferences on reproductive care services.

### Limitations

Our study has some limitations. First, notwithstanding the saturation observed in our interviews, we may have still missed out important categories that could have emerged among stakeholders in our study. We also acknowledge the possibility of health staff and policy implementer’s biases in trying to withhold information on the study aim. Our triangulation of views have minimizes potential errors on stakeholder narratives. We acknowledge the inability of our study to investigate views from persons at the health ministry level and other public-private partners involved in implementing reproductive health strategies. Nevertheless, the three group of stakeholders examined in this study represent a good fit when examining district level stakeholders on sexual and reproductive care needs in any rural context in Ghana.

## Conclusion

This study explored stakeholder’s views on experiences, expectations, and decision making on reproductive care among rural Ghanaian women. Gender mainstreaming approaches in Ghana should develop structures and systems within the health delivery system that will create conditions of mutual respect among provider-user, and empower women in their healthcare seeking behaviors. In addition, health teams working to improve sexual and reproductive health care must find suitable context strategies that will effectively work to improve women reproductive care needs at their operational levels. From our results, accounts of services non-differentiation of services among adolescents and older women group’s calls for the need for health providers at facility levels to adjust health services to target and meet specific needs for different user groups. Whiles we support the inclusion of post-abortion care and post-pregnancy termination management issues into the National health insurance benefit package, a broad program scheme that ensure sexual and reproductive health needs are truly subsidized or provided essentially at lower fee cost will benefit rural population groups.

To support accountability and equity in reproductive services provision at facility levels, the local health systems need to be resourced to evaluate health staff working attitudes in relation with user needs and satisfied reproductive care demands. To upscale services using the private sector contributor approach, policies that target increased private sector participation may result in more multi-faceted reproductive services care provisions, something not entirely available in most public sector health facilities.

Health sector leads such as the Ministry of Health and Ghana Health Service should drive the need to close the inclusiveness gap on sexual and reproductive care sector policy planning processes and needs. Stakeholder consensus from this study also advocates strongly for clear guidelines on delivery reproductive care to be contextualized to meet user care needs. Health counseling and the need for individualized care have increasingly been evidenced as an entry approach to reach new users of health services. To fully reach individualized care at community level, health systems must have community support clutches such as the mother-to-mother support groups evidenced from our study. This is important to provide community level psychological support and delimit cluttered relationships that may exist between provider-user during formal facility access hours.
